# Betaine reduces β-amyloid-induced paralysis through activation of cystathionine-β-synthase in an Alzheimer model of *Caenorhabditis elegans*

**DOI:** 10.1186/s12263-018-0611-9

**Published:** 2018-07-27

**Authors:** Anne Leiteritz, Benjamin Dilberger, Uwe Wenzel, Elena Fitzenberger

**Affiliations:** Molecular Nutrition Research, Interdisciplinary Research Center, Justus-Liebig-University of Giessen, Heinrich-Buff-Ring 26-32, 35392 Giessen, Germany

**Keywords:** Alzheimer’s disease, β-Amyloid, *Caenorhabditis elegans*, Hyperhomocysteinemia, Betaine

## Abstract

**Background:**

The neurodegenerative disorder Alzheimer’s disease is caused by the accumulation of toxic aggregates of β-amyloid in the human brain. On the one hand, hyperhomocysteinemia has been shown to be a risk factor for cognitive decline in Alzheimer’s disease. On the other hand, betaine has been demonstrated to attenuate Alzheimer-like pathological changes induced by homocysteine. It is reasonable to conclude that this is due to triggering the remethylation pathway mediated by betaine-homocysteine-methyltransferase. In the present study, we used the transgenic *Caenorhabditis elegans* strain CL2006, to test whether betaine is able to reduce β-amyloid-induced paralysis in *C. elegans*. This model expresses human β-amyloid 1–42 under control of a muscle-specific promoter that leads to progressive, age-dependent paralysis in the nematodes.

**Results:**

Betaine at a concentration of 100 μM was able to reduce homocysteine levels in the presence and absence of 1 mM homocysteine. Simultaneously, betaine both reduced normal paralysis rates in the absence of homocysteine and increased paralysis rates triggered by addition of homocysteine. Knockdown of cystathionine-β-synthase using RNA interference both increased homocysteine levels and paralysis. Additionally, it prevented the reducing effects of betaine on homocysteine levels and paralysis.

**Conclusion:**

Our studies show that betaine is able to reduce homocysteine levels and β-amyloid-induced toxicity in a *C. elegans* model for Alzheimer’s disease. This effect is independent of the remethylation pathway but requires the transsulfuration pathway mediated by cystathionine-β-synthase.

## Background

Alzheimer’s disease (AD) is the most common age-related neurodegenerative disorder caused by the accumulation of aggregated β-amyloid (Aβ) as senile plaques in the human brain [1, 2]. These deposits are associated with synaptotoxicity that leads to the characteristic cognitive decline [3]. Moderately elevated plasma homocysteine (Hcy) levels were identified as a strong risk factor not only for vascular dementia but also for AD [4]. Several mechanisms underlying the noxious effect of Hcy in the brain have been proposed. These include DNA damage [5], activation of *N*-methyl-d-aspartate receptors [6], and the alteration of the amyloid precursor protein (APP) metabolic pathway by hypomethylation [7]. Slightly increased homocysteine levels can be due to single nucleotide polymorphisms in the gene encoding 5,10-methylene-tetrahydrofolate-reductase (MTHFR) [8]. It has been shown that in such cases folic acid supplementation reduces Hcy levels [9]. Alternatively, remethylation of Hcy to methionine and S-adenosylmethionine (SAM) can also be achieved by supplementing betaine as a methyl donor [10]. Indeed, in hyperhomocysteinemic rats betaine supplementation was shown to ameliorate Hcy-induced AD-like pathological changes and memory deficits [11]. In the nematod*e Caenorhabditis elegans*, however, effects of betaine on remethylation of Hcy can be excluded since the nematode has no orthologue of betaine-Hcy-methyltransferase (BHMT), encoding the relevant enzyme for betaine mediated remethylation [12]. Another biochemical pathway, through which Hcy can be detoxified, is transsulfuration of Hcy to cystathionine by the vitamin B6-dependent enzyme cystathionin-β-synthase (CBS).

In the present study, we used the transgenic *C. elegans* strain CL2006, which expresses the human Aβ under the control of the muscle-specific *unc-54* promotor and displays progressive paralysis [13], in order to test the effects of betaine supplementation on the AD phenotype. To explore the contribution of CBS on the measured effects, RNA interference was used. Moreover, the effects of betaine intervention and CBS knockdown on Hcy levels were estimated by ELISA.

## Methods

### Strains

The transgenic nematode strain CL2006 ((*dvIs2*[pCL12(*unc-54*/human Aβ_1–42_ minigene) + pRF4]) was obtained from the *C. elegans* Genetics Center, CGC (University of Minnesota, MN, USA). CL2006 expresses human Aβ_1–42_ under the control of the muscle-specific promoter *unc-54*, leading to progressive, adult-onset paralysis [13]. *E. coli* HT115 RNAi clones were purchased from Source Bioscience (Cambridge, UK) and included a negative control (empty L4440 vector), *cbs-1* (ZC373.1), *gcs-1* (F37B12.2), and *metr-1* (R03D7.1).

### *C. elegans* maintenance

The maintenance and experimental procedures were conducted according to standard protocols. The nematodes were cultivated at 20 °C on nematode growth medium (NGM) agar plates that contained an *E. coli* OP50 lawn as the major food source [14]. For the experiments, synchronous populations of larvae were used, which were obtained by bleaching egg-laying adults with a hypochlorite solution [15].

### RNA interference

RNA interference (RNAi) was performed by using the feeding method. As described elsewhere, it was conducted in liquid cultures, which were enriched with RNAi bacteria [16, 17]. In brief, in the RNAse III-deficient *E. coli* strain HT115 gene-specific dsRNA expression was induced by incubation with 1 mM isopropyl-β-d-thiogalactopyranoside for 4 h at 37 °C. Subsequently, the bacteria were centrifuged and resuspended in NGM, containing 50 μg/ml kanamycin to inactivate bacterial growth. This suspension was applied to each well of a 96-well plate together with a volume of 10 μl M9 buffer containing 10–15 synchronized L1 larvae. The volume of the NGM-bacteria-suspension varied between 44 and 46 μl, depending on the number of effectors, which were added later. That is, in case of betaine supplementation only, 44 μl NGM-bacteria suspension were used, and if Hcy and betaine were applied together, 46 μl NGM were added.

### Treatment of *C. elegans* with Hcy and betaine

To young adult CL2006 nematodes, which are characterized by the ability to lay eggs, a volume of 6 μl betaine or 7 μl of both betaine and homocysteine were added. Effector solutions were prepared in M9 buffer and contained tenfold enriched concentrations compared to the final concentration in the experiment. Control nematodes were always treated with the identical volumes of M9 buffer only. Treatments were performed for 48 h before heat-shock was applied.

### Measurement of paralysis

The expression and aggregation of Aβ_1–42_ in *C. elegans* results in the paralyzation phenotype, which is described as a movement that is restricted to waving of the head without translocation of the animal [18].

Before scoring paralysis, the nematodes were heat-shocked for 1.5 h at 35 °C in order to accelerate the paralyzation process and to generate a paralyzation rate in the control population of about 50%. After heat shock, the individual nematodes were transferred with M9/Tween 20®- buffer on NGM agar plates. The paralysis phenotype was examined by visual analysis of 25 nematodes per treatment group. Each nematode was tapped with a bent platinum wire, and subsequently, its moving ability was scored.

### Hcy-ELISA

For determination of Hcy concentrations, a quantitative, competitive human Hcy-ELISA Kit (NeoScientific, Cambridge; USA) was employed. To this end, nematodes were treated with lysis buffer (HEPES 50 mM, NaCl 150 mM, EDTA 5 mM, DTT 2 mM) and frozen at − 80 °C to extract proteins. After thawing on ice, worms were homogenized with Peqlab Precellys 24-Dual (VWR, Erlangen, Germany). Subsequently, the concentration of extracted protein was determined by using the Bio-Rad Protein Assay. The measurement of the Hcy concentration was set up according to the instruction manual. In brief, the protein solution and a Hcy-HRP conjugate were added to the wells of the ELISA plate, which were coated with a Hcy-specific antibody. Hcy in the samples competes with the conjugate for binding to the plate-bound antibody. Higher Hcy concentrations in the probes lead to decreased Hcy-HRP conjugate binding. By using a HRP substrate, the amount of captured Hcy-HRP can be quantified colorimetrically. The OD of the probes was compared to a standard curve, which was generated by using the included calibration standards.

### Calculations and statistics

For statistical analyses, GraphPad Prism 5.0 software (GraphPad, La Jolla, CA, USA) was used. Analysis of variance (ANOVA) and Bonferroni-Holm multiple comparison test as well as 2-way ANOVA were performed for multiple group comparisons. Differences between two groups were examined with Student’s *t* test. The results shown are representatives of at least three independent experiments and are presented as means ± SD. Significance levels were assumed as *p* < 0.05 (*), *p* < 0.01 (**), and *p* < 0.001 (***).

## Results

### Knockdown of *cbs-1* increases Hcy levels and paralysis in CL2006

**Fig. 1:**
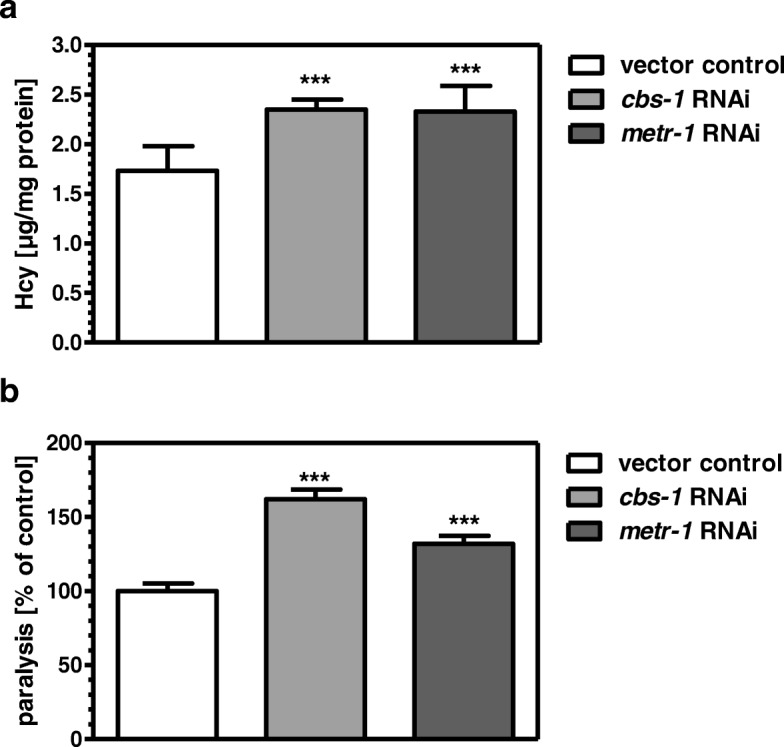
Knockdown of *cbs-1* and *metr-1* via RNAi increase Hcy level and paralysis rate in CL2006. Hcy concentration was determined with the Human Hcy-ELISA Kit 48 h after nematodes had reached the young adult stage (**a**). At the same, stage paralysis in CL2006 was assessed subsequent to a heat-shock for 1.5 h at 35 °C by tapping the nematodes with a bent platinum wire and subsequent scoring of their moving ability (**b**). ***p < 0.001 versus the vector control

In order to test whether one-carbon metabolism is functional in *C. elegans*, we knocked down the genes for *cbs-1* and and 5-methyltetrahydrofolate-methyltransferase *metr-1*, which is synonymous for methionine synthase. Knockdown of either gene caused an increase of Hcy levels (Fig. 1a). These results suggest that, with regard to Hcy remethylation and transsulfuration, both enzymes possess the same functional role in *C. elegans* and in humans. Moreover, both knockdowns significantly increased the paralysis rate in CL2006 (Fig. 1b).

### Hcy levels are directly associated with the paralysis phenotype in CL2006

**Fig. 2:**
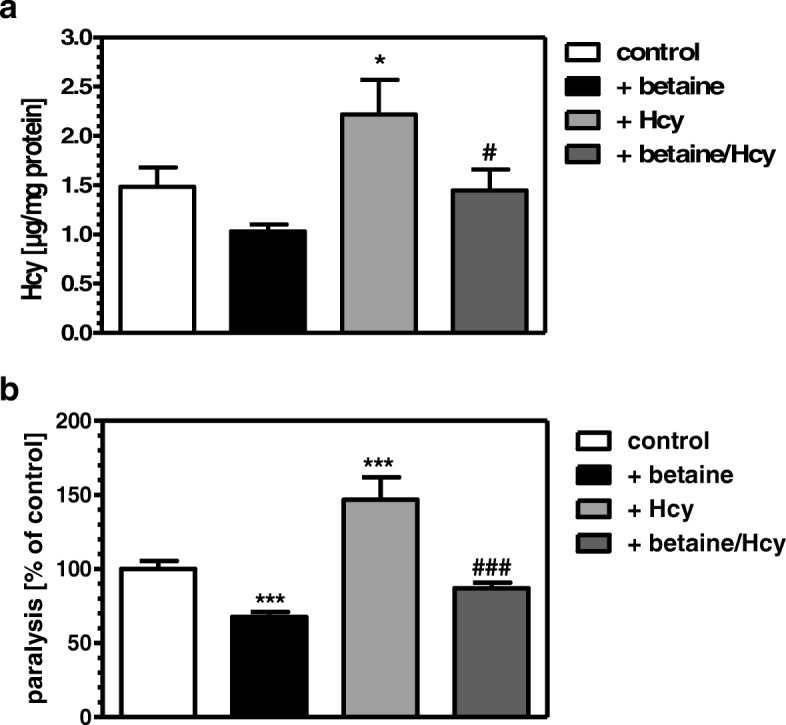
Betaine reduces Hcy level and paralysis rate in the absence and presence of exogenous Hcy. Hcy level was determined either in control nematodes or those treated with 1 mM Hcy. Moreover, in an additional experiment, both groups were exposed to 100 μM betaine also (**a**). Paralysis was determined under identical conditions as described for Hcy measurements (**b**). *p < 0.05, ***p < 0.001 versus the control; ^###^*p* < 0.001 versus worms treated with Hcy

Next, we assessed whether the observed paralysis under knockdown of *cbs-1* and *metr-1* are indeed due to the increased Hcy levels. To this end, 100 μM betaine was applied in the absence or presence of 1 mM exogenous Hcy. Although the reduction of Hcy levels by betaine treatment was not significant, betaine was able to significantly reduce Hcy levels after application of 1 mM exogenous Hcy (Fig. 2a). Significant interactions of homocysteine and betaine were observed (p < 0.001).Moreover, paralysis rate decreased upon betaine treatment both in the absence of exogenous Hcy and when paralysis was first increased by addition of Hcy to the nematodes (Fig. 2b).

### Betaine does no longer affect paralysis under *cbs-1* RNAi

**Fig. 3:**
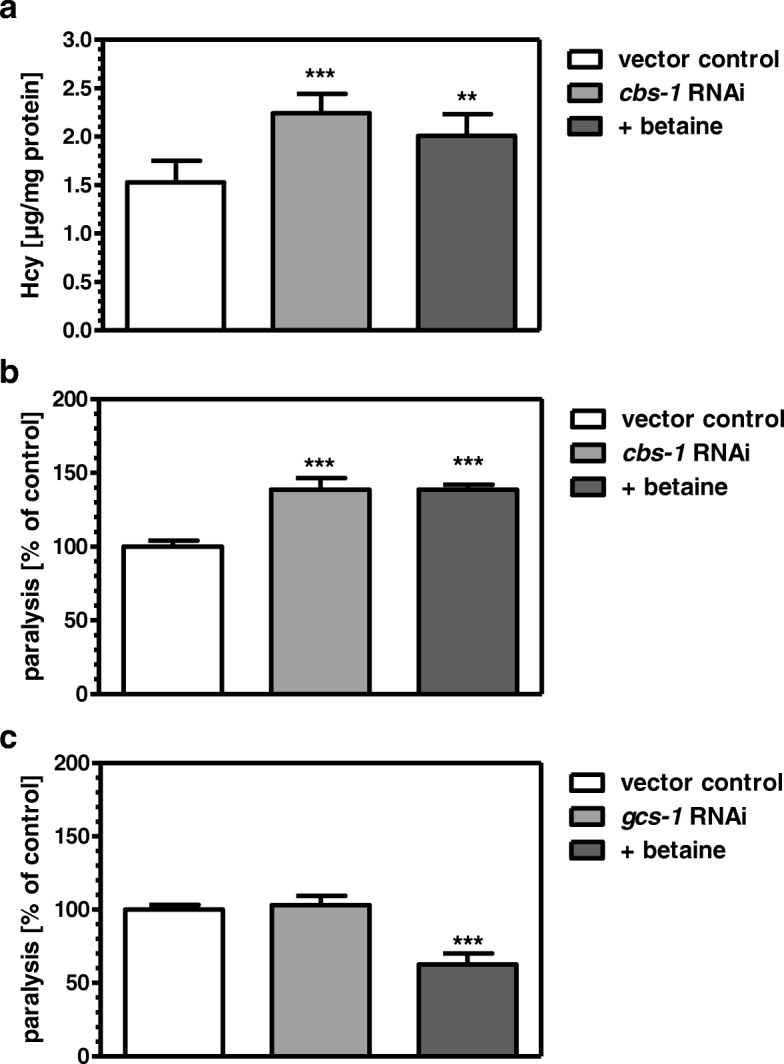
The glutathione synthesis is not involved in the paralysis reducing effects of betaine. Under *cbs-1* RNAi, betaine was no longer able to reduce Hcy level significantly (**a**) nor to diminish paralysis (**b**). Knockdown of *gcs-1*, encoding the key enzyme for glutathione synthesis, did neither affect paralysis nor prevent the paralysis reducing effects of betaine (**c**). **p < 0.01, ***p < 0.001 versus vector control

In order to find out whether the paralysis-reducing effects of betaine depend on the activity of CBS, we knocked down the corresponding gene again. RNAi for *cbs-1* completely prevented the reducing effect of betaine on Hcy levels (Fig. 3a) and paralysis (Fig. 3b). These findings suggest that, on the one hand, betaine acts via triggering the transsulfuration pathway. On the other hand, increased transsulfuration of Hcy would provide more cysteine, which could serve as a substrate for glutathione synthesis. This could be of special importance in preventing the AD-phenotype, since AD is frequently shown to be associated with increased oxidative stress. However, when we knocked down *gcs-1 (*γ-glutamyl-cysteine-synthetase*)*, encoding the key enzyme for glutathione synthesis, we did not observe any influence on the paralysis rate. Moreover, betaine was still able to reduce paralysis in CL2006 to the same extent as in the presence of GCS-1 (Fig. 3c).

## Discussion

Senile plaques consisting of Aβ are a hallmark of AD and are considered as responsible for neuronal damage [19]. It is suggested that Aβ oligomers are neurotoxic because they lead to increased oxidative stress [20], mitochondrial dysfunction [21], disturbed metal ion homeostasis [22], and apoptosis [23]. Hyperhomocysteinemia has been shown to be associated with AD and represents a factor which is influenced genetically and environmentally [24]. The impact of genetic impairments of one-carbon metabolism, as is case by MTHFR C677T polymorphism, prevails especially under insufficient folate, vitamin B6, and/or vitamin B12 levels [24]. Increased Hcy levels, as a consequence of insufficient remethylation and/or transsulfuration, have been described to increase the production of reactive oxygen species via autoxidation of homocysteine [25]. In addition, homocysteine and the oxidized metabolite homocysteic acid are described as potent neurotoxins because of its property to be an endogenous NMDA (*N*-methyl-d-aspartate) receptor agonists [6]. High levels of homocysteine also are associated with DNA damage because the amino acid impairs the DNA repair, supporting neuronal cell death [5]. These processes increase indirectly the vulnerability of neurons to get damaged by Aβ [26, 27]. There is, moreover, also a direct effect of increased Hcy concentrations on Aβ toxicity, which is due to the enhanced aggregation propensity of Aβ_1–42_ via the homocysteinylation of lysine residues leading to stabilized soluble oligomeric intermediates [28].

Nutritional factors able to decrease Hcy levels and to cause slowing of cognitive decline and of atrophy in critical brain regions, consistent with modification of the AD process at least in high risk subjects with baseline vitamin B status, are folate, vitamin B6, and vitamin B12 [4]. Whereas vitamin B6 could increase the synthesis of cystathionine from Hcy and serine by CBS, folate as cofactor for MTHFR and B12 for methionine-synthase might increase under those conditions remethylation of Hcy, betaine could serve also as methyl group donor for Hcy as mediated by BHMT. Indeed betaine has been shown in this context to arrest Hcy-induced AD-like pathological changes and memory deficits more efficient than supplementation of folate [11]. In *C. elegans*, however, BHMT is not present due to genetic loss [12]. Accordingly, *C. elegans* represents the perfect model to investigate whether there are effects of betaine on AD-like phenotypes which are independent on the remethylation of Hcy.

In *C. elegans* CL2006, expressing a transgene for human Aβ, inhibition of remethylation by *metr-1* RNA, or transsulfuration by *cbs-1* RNAi both increased Hcy levels and paralysis. This demonstrates that one-carbon metabolism is functional also in *C. elegans* as has been observed previously with regard to adaptive responses to dietary restriction [29]. Moreover, the results suggest that Hcy levels are directly associated with the paralytic phenotype in CL2006. The latter was substantiated by the findings that increased Hcy levels by addition of exogenous Hcy lead to increased paralysis whereas lowering of Hcy levels in the presence or absence of exogenous Hcy reduces the paralysis rate to a similar extent at the same dose. Although it might be concluded that lowering of Hcy levels by betaine could be due to activation of MTHFR and methionine-synthase. We did not perceive it as rationale to follow this possibility since enhanced levels of SAM would allosterically activate CBS [30]. We therefore focused on CBS as a target for the observed betaine effects. Indeed betaine was no longer able to reduce Hcy levels or paralysis when increased by *cbs-1* RNAi. We finally postulated that increased Hcy detoxification under betaine exposure by increased cystathionine synthesis could, through cleavage by cystathionase, deliver more cysteine for glutathione synthesis. As described above, enhanced generation of reactive oxygen species is a typical phenomenon of AD and has been attributed to decreased levels of the brain antioxidant, glutathione [31]. Moreover, lowered cortical glutathione levels were found as a biomarker of early Alzheimer disease pathogenesis [32]. However, in CL2006, the knockdown of *gcs-1*, encoding the rate-limiting enzyme for glutathione synthesis, did neither affect the paralysis rate nor did it prevent betaine from being active in diminishing paralysis.

## Conclusions

In conclusion, our study shows that betaine reduces the Aβ-induced degeneration in an Alzheimer model of the nematode *C. elegans* by lowering the Hcy levels. CBS was, moreover, identified as the target enzyme through which betaine exerts its degeneration preventing effects.

## Abbreviations

AD: Alzheimer’s disease
Aβ: β-Amyloid
BHMT: Betaine-homocysteine-methyltransferase
CBS: Cystathionine-β-synthase
GCS: γ-Glutamyl-cysteine-synthetase
Hcy: Homocysteine
HRP: Horseradish peroxidase
METR: 5-Methyltetrahydrofolate-homocysteine-methyltransferase
MTHFR: 5,10-Methylene-tetrahydrofolate-reductase
SAM: *S*-Adenosylmethionine
